# A consensus map for quality traits in durum wheat based on genome-wide association studies and detection of ortho-meta QTL across cereal species

**DOI:** 10.3389/fgene.2022.982418

**Published:** 2022-08-30

**Authors:** Ilaria Marcotuli, Jose Miguel Soriano, Agata Gadaleta

**Affiliations:** ^1^ Department of Agricultural and Environmental Science, University of Bari Aldo Moro, Bari, Italy; ^2^ Sustainable Field Crops Programme, IRTA (Institute for Food and Agricultural Research and Technology), Lleida, Spain

**Keywords:** candidate genes, grain quality, synteny, *Brachypodium*, rice, maize

## Abstract

The present work focused on the identification of durum wheat QTL hotspots from a collection of genome-wide association studies, for quality traits, such as grain protein content and composition, yellow color, fiber, grain microelement content (iron, magnesium, potassium, selenium, sulfur, calcium, cadmium), kernel vitreousness, semolina, and dough quality test. For the first time a total of 10 GWAS studies, comprising 395 marker-trait associations (MTA) on 57 quality traits, with more than 1,500 genotypes from 9 association panels, were used to investigate consensus QTL hotspots representative of a wide durum wheat genetic variation. MTA were found distributed on all the A and B genomes chromosomes with minimum number of MTA observed on chromosome 5B (15) and a maximum of 45 on chromosome 7A, with an average of 28 MTA per chromosome. The MTA were equally distributed on A (48%) and B (52%) genomes and allowed the identification of 94 QTL hotspots. Synteny maps for QTL were also performed in *Zea mays*, *Brachypodium*, and *Oryza sativa*, and candidate gene identification allowed the association of genes involved in biological processes playing a major role in the control of quality traits.

## Introduction

Cereals are the main species in Mediterranean cropping systems, well adapted to semi-arid climate conditions and able to give a stable economic sustain to the farmers. Wheat represents a target crop for Mediterranean agriculture and provides 20% of calories to the world population, highlighting the relevance of this crop for current and future strategic cultivation (Royo et al., 2017). In this context, we must intensify efforts toward crop improvement and yield stability under conditions of sustainable agricultural production.

Durum wheat (*Triticum turgidum* ssp. durum) is largely produced in the Mediterranean basin being used for human nutrition, prevalently transformed into semolina for pasta and couscous, but it can also be used to obtain flour for bread (Kadkol and Sissons, 2016). Due to the ongoing climate changes and steady increase in average temperatures, the flour quality of commercial genotypes may no longer be able to perform well in the coming years. The importance of grain quality parameters for durum wheat end-products in the food chain makes it a crucial tool in maintaining or increasing durum wheat production under disease pressure and adverse climatic conditions to preserve the grain quality (Beres et al., 2020).

Since crop quality is a complex trait, it can be either related to end-use properties or to nutritional content and these parameters are regulated by different compounds, the genetic determination of quality traits is rather complicated. Due to the high environmental influence on crop quality traits, the search for genes related to them is more complicated such as the transfer of these characters, despite the presence of novel genomic tools (Yang et al., 2022).

The research activities for wheat quality improvement have been focused in the last decade on the valorization of wheat germplasm collections including old varieties and wild relatives besides the obtainment and evaluation of new breeding lines. Several studies have been conducted on the environmental effects on quality, development of evaluation methods, and processing for end-users (Pour-Aboughadareh et al., 2021). Therefore, the nutritional improvement of crops has a positive impact on millions of people around the world without the need to alter their eating habits. Currently, the aspects related to human health have taken great relevance in cereal breeding programs to develop biofortified crops (Swamy et al., 2021).

The increasing awareness of the use of cereal-based products in a healthy diet is currently becoming more and more evident, and so plenty of studies focus on the identification and exploitation of natural variations of bioactive components in the grain. Wheat has a protein content of about 13% and is the leading source of vegetable protein in human food, and it is also an important source of carbohydrates. When wheat is eaten as a whole grain, it is an excellent source of dietary fiber and nutrients (Marcotuli et al., 2020). Many of the abovementioned traits are polygenic traits associated with quantitative trait loci (QTL) located on all the tetraploid wheat chromosomes. To identify main effective genes for wheat quality is a main target of wheat breeders worldwide, however, the efficiency of selection is constrained by the following: 1) the decline of genetic diversity in elite germplasm (by the pursuit of elite high-performing cultivars) that leads to scarcity or even absence of suitable loci in modern breeding lines; 2) limited knowledge on quality traits that are often complex QTL influenced by numerous genes and environmental conditions; 3) lack of adequate molecular knowledge to lay the foundation for molecular breeding. These limitations are more severe in durum wheat with respect to bread wheat. The underlined problem can be solved with the use of high-density genetic maps, new molecular markers, or a wide collection summarized in GWAS analyses.

GWAS detects the association between genotype and trait of interest using conserved linkage disequilibrium (LD) present in a selected panel of accessions (Myles et al., 2009). Recently, association or LD mapping, utilizing genome-wide markers, has been adopted in wheat because of two main advantages: 1) association mapping does not require the cost and time associated with the population and genetic map development, and 2) GWAS provides high mapping resolution as it efficiently uses the multiple historical crossover events occurred in the diverse association panel used (Saini et al., 2022).

GWAS give, also, information about MTAs that could be utilized for candidate gene discovery and characterization adoptable in breeding programs. A study from Saini et al. (2022) underlines that among 86,122 wheat lines studied under various GWAS analyses 46,940 loci associated with traits were reported, but further utilization of these markers was largely limited. To solve this situation QTL hotspots (genomic locations enriched in QTL) are a common and notable feature when collecting many QTL for various traits in many areas of biological studies (Wu et al., 2021). This approach is a good instrument to study at the same time many traits, trying to find the consensus and most robust QTL using the information reported in multiple studies for the reliability of their location and effect across different genetic backgrounds and environments, as well as to refine QTL positions on a consensus map (Goffinet and Gerber, 2000). The approach developed by these authors is called QTL meta-analysis and it allows the identification of the genome regions most involved in trait variation, being a suitable approach to narrow down QTL regions, to tackle map-based cloning strategies, and to identify candidate genes (Soriano and Alvaro, 2019; Wu et al., 2021). Regarding GWAS, a statistical framework for QTL hotspot detection was reported by Wu et al. (2021), allowing the integration of independent GWAS studies in a reference map for durum wheat. The use of MTA in meta-analysis represents an important and complementary tool for the identification of new stable Genome-wide QTL hotspots and the discovery of new alleles for durum wheat quality. The most powerful hotspots have also been used for the detection of the so-called ortho-Meta-QTL, which are conserved among species and hence will be more reliable and can be used in different cereals.

In the present manuscript, MTA detected by GWAS were projected for the first time on the durum wheat consensus map from Maccaferri et al. (2019) allowing the identification of QTL hotspots related to quality traits in durum wheat, genes directly associated and controlling different quality traits and regulatory genes, and finally conserved orthologous regions among bread wheat, durum wheat, rice, maize, and *Brachypodium*.

## Materials and methods

### Quality traits and marker-trait association database

Genome-wide association studies for quality traits were retrieved using the keywords “durum wheat quality GWAS” from the Web of Science server (http://apps.webofknowledge.com). The marker-trait association (MTA) for the GWAS studies database was created from 10 studies published from 2015 to 2020 (Table 1). The database reported information on the diversity panel used, number of genotypes, number of MTA, and traits analyzed in each study. A total of 57 different traits were collected from the GWAS studies (Table 2). The MTA database reported information on the name of the chromosome and position of the MTA, the confidence interval (CI), and the phenotypic variance explained (PVE) by each MTA.

**TABLE 1:** Summary of QTL studies included in the meta-analysis.

References	Panel	Size	N MTA	Traits
Marcotuli et al. (2015)	Agrogen	104	19	AX
Marcotuli et al. (2016)	Agrogen	230	7	BG
Colasuonno et al. (2017)	Durum collection (7 subspecies)	124	6	YPC
Reimer et al. (2008)	Worldwide elite durum wheat	93	20	YPC
Rosello et al. (2018)	Mediterranean landraces	172	14	GPC, GS, TW, YPC
Johnson et al. (2019)	Cultivars and inbred lines (1997–2014)	243	163	CLOSS, Color, CWT, dif, FIRM, GLUT, GPC, GS, MIXO, PPO, SASH, SEXT, SPROT, SV, TEXT, VIT, WG, WTS, YPC
Taranto et al. (2020)	Italian durum breeding lines	79	44	Gli, Gli + Glu, HMW/LMW, IP, TPT
Alemu et al. (2020)	Ethiopian durum wheat	192	20	a*, b*, L*, GL, GW
N'Diaye et al. (2018)	Canadian durum breeding lines	192	80	DEE, DOE, DOTE, GPC, GS, PLOSS, PRLOSS, YPC
N'Diaye et al. (2017)	Canadian durum wheat collection	169	22	PLOSS, YPC

**TABLE 2:** Traits analyzed for QTL meta-analysis.

Trait	Description	Trait	Description
a*	Redness	GSC	Grain sulfur content
AX	Arabinoxylan	GSeC	Grain selenium content
b*	Yellowness	GseY	Grain selenium yield
BG	β-glucan	GW	Grain width
CLOSS	Cooking loss	GZnC	Grain zinc content
Color	Color a, b, or L	HMW	High molecular weight GS
Color	Pasta color	IP	Immunogenic gluten epitopes
CWT	Cooked weight	L*	Brightness
DEE	Deformation energy	LMW	Low molecular weight GS
dif	Difference in color a, b or L	MIXO	Mixogram score
DOE	Dough extensibility	PGC	Phosphorus grain content
DOTE	Dough tenacity	PLOSS	Pigment loss
Fb	Flour yellow color	PPO	Polyphenol oxidase activity
FIRM	Firmness	PRLOSS	Protein loss
GCaC	Grain calcium content	SASH	Semolina ash
GCdC	Grain cadmium content	SEXT	Semolina extraction
GCuC	Grain copper content	SPROT	Semolina protein
GFeC	Grain iron content	SV	SDS-sedimentation volume
GKC	Grain potassium content	TEXT	Total extraction
GL	Grain length	TPT	Toxic peptides
Gli	Gliadins	TW	Test weight
Glu	Glutenins	VIT	Kernel vitreouness
GLUT	Glutork	WG	Wet gluten
GMgC	Grain magnesium content	WTS	Work to shear
GMnC	Grain manganese content	YPC	Pasta b*
GPC	Grain protein content	YPC	Semolina b*
GPC	Protein content	YPC	Semolina pigment
GS	Gluten index	YPC	Yellow pigment content
GS	Gluten strength		

### Projection on the consensus map

To represent all the MTA in the same linkage map, the durum wheat consensus map developed by Maccaferri et al. (2015) was used for projection following the homothetic approach described by Chardon et al. (2004) as previously described in Colasuonno et al. (2021). When the CIs were not reported in the original studies, they were estimated according to the linkage disequilibrium (LD) decay for each chromosome.

### Identification of quantitative trait loci hotspots

To simplify the MTA information, the associations were grouped into QTL hotspots. First, the CIs were standardized using the formula described by Chardon et al. (2004):

$$S_{i}^{2}={\left(\frac{\text{CI}}{3.92}\right)}^{2}$$

where CI corresponded with the original CI or the LD decay for each chromosome. To define a hotspot, the density of MTAs along the chromosome was calculated as the QTL overview index (Chardon et al., 2004) for each cM of the genetic map reported by Maccaferri et al. (2015):

$$U=\frac{\frac{nbQTL}{nbE}}{Total\;length\;of\;map}$$

where nbQTL is the number of MTA and nbE is the total number of studies.

Breeding QTL hotspots were selected based on three criteria according to Löffler et al. (2009): small supporting intervals of the mQTL, high number of initial QTL, and high phenotypic variance explained (PVE) by the initial QTL. In our case, we considered for QTL hotspots a CI lower than 20Mb, and subsequently, a PVE > 0.04 when 5 or more MTAs per hotspot were reported or PVE > 0.1 when MTAs per hotspots was 3. QTL hotspot distribution was compared among the genetic and physical maps of durum wheat (Maccaferri et al., 2015; Maccaferri et al., 2019). Chromosomes were equally distributed in five bins based on their genetic and physical length. Subsequently, the total number of QTL hotspots per bin was counted.

### Synteny analysis and identification of candidate genes underlying the MQTL

A total of 12,606 markers associated with QTL for quality from *Triticum* ssp. were palmed on the genomes of *O. sativa*, Z*. mays,* and *B. distachyon* to extract the syntenic positions of the markers in the three genomes using a mapping approach and draw a circus diagram for data view for each analysis. The following reference genomes were used: *B. distachyon* version 1 (http://www.plantgdb.org/BdGDB/); *O. sativa Japonica* Group version IRGSP-1.0 (http://rice.uga.edu/); and *Z. mays* version AGPv3 (https://www.maizegdb.org/).

An ad-hoc mapping pipeline was developed to map the markers and highlight all the syntenic positions between *Triticum* spp. and three different monocot genomes. The core algorithm of this pipeline was the bwa-mem aligner, and the workflow was divided into three parts: 1) perform three different mappings for each genome with different quality parameters, from higher to lower stringency; 2) merge the results of the five mapping steps considering several quality parameters including the redundancy of the alignments, the quality of the markers, and the quality of the alignments; 3) filter by mapping length >60%, which means to keep all the hits with a mapping length >60% with respect to the marker sizes, and a spread-scaled quality >10, which measures the posterior probability that the mapping position is wrong.

Gene models within syntenic QTL hotspot were identified using the high-confidence genes reported for the Svevo durum wheat reference sequence, available at https://wheat.pw.usda.gov/GG3/jbrowse_Durum_Svevo, and the bread wheat Chinese spring reference sequence, available at https://wheat-urgi.versailles.inra.fr/Seq-Repository. Homologous genes from “Chinese Spring” and “Svevo” were subsequently identified in the syntheny region of Maize (https://www.maizegdb.org/), Rice (http://rice.uga.edu/), and *Brachypodium* (http://www.plantgdb.org/BdGDB/) databases.

Circular figures of the QTL hotspots and chromosome synteny were created using the online software Clico FS (Cheong et al., 2015) available at http://clicofs.codoncloud.com.

## Results

### Marker-trait association distribution and quantitative trait loci hotspots identification

**FIGURE 1:**
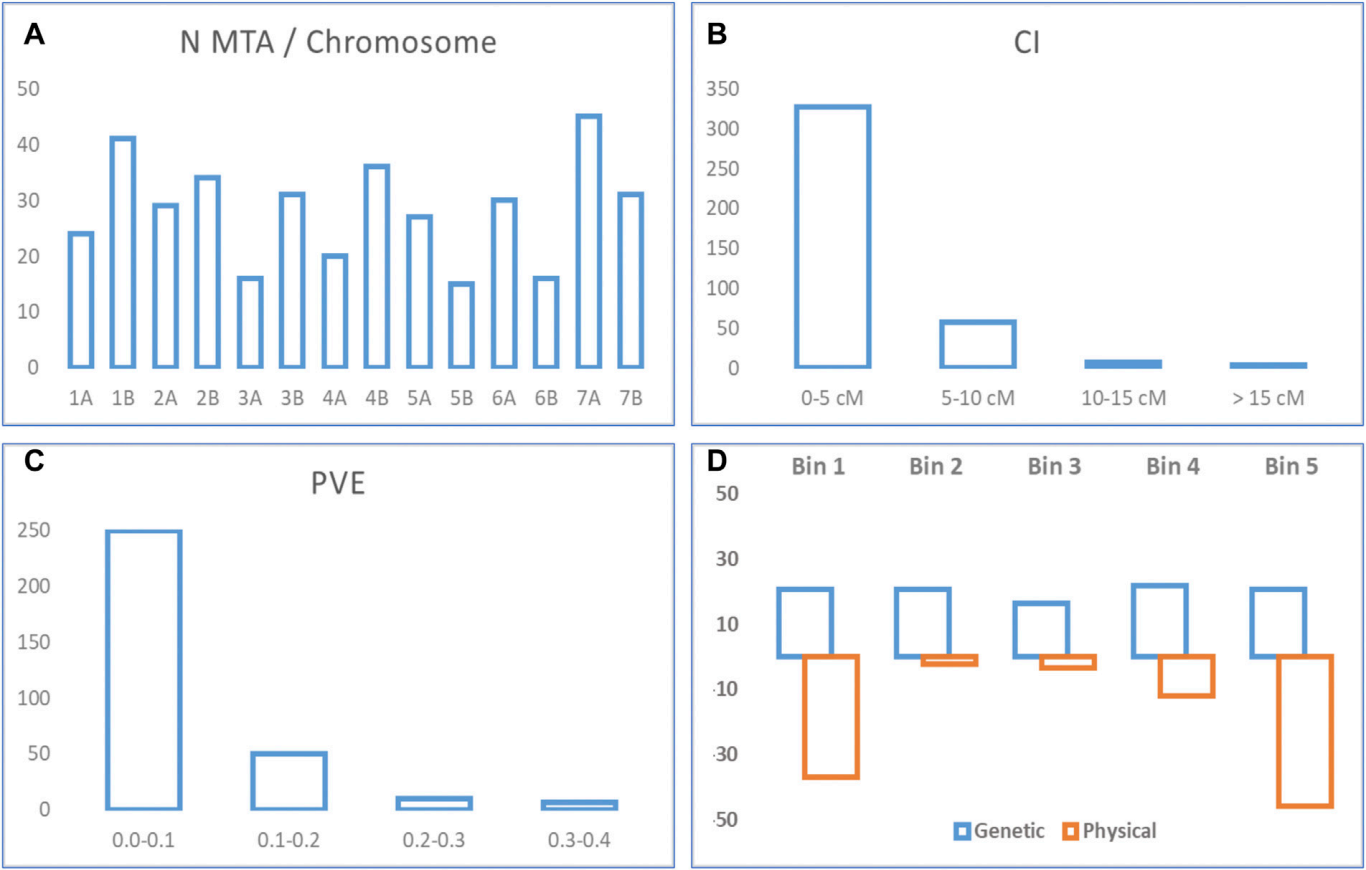
MTA statistics. **(A)** Number of MTA per chromosome. **(B)** Confidence interval from original MTA. **(C)** PVE from original MTA. **(D)** Percentage of QTL hotspots per bin in the genetic and physical maps.

Ten studies published from 2015 to 2020 reporting 395 MTA for quality traits were collected (Table 1; Supplementary Table S1). The studies covered a total of 1,598 genotypes (including elite cultivars, breeding lines, and landraces). MTA was distributed throughout the 14 chromosomes (A and B genomes) of durum wheat. The number of MTA per chromosome ranged from 15 on chromosome 5B to 45 on chromosome 7A, with an average of 28 MTA per chromosome. Forty-eight percent of the MTA were identified in genome A, and 52% in genome B (Figure 1A). When the trait was considered, out of 39 traits, yellow pigment content (YPC) presented the highest number of MTA (24%). Confidence intervals (CI) ranged from 0.1 to 43 cm with an average of 3.3 cm. For 203 MTA not reporting a CI in the original study, the intra-chromosomal LD decay was used to establish their CI. Most of the MTA (97%) showed a CI lower or equal to 10 cm, whereas the CI for 83% of the MTAs was lower or equal to 5 cm (Figure 1B). The proportion of the phenotypic variance explained (PVE) followed a typical quantitative inheritance as previously reported in other QTL meta-analysis studies (Soriano and Alvaro, 2019; Soriano et al., 2021). 79% of the MTA showed a PVE lower than 0.1, whereas it increases to 95% for a PVE < 0.2 (Figure 1C).

**FIGURE 2:**
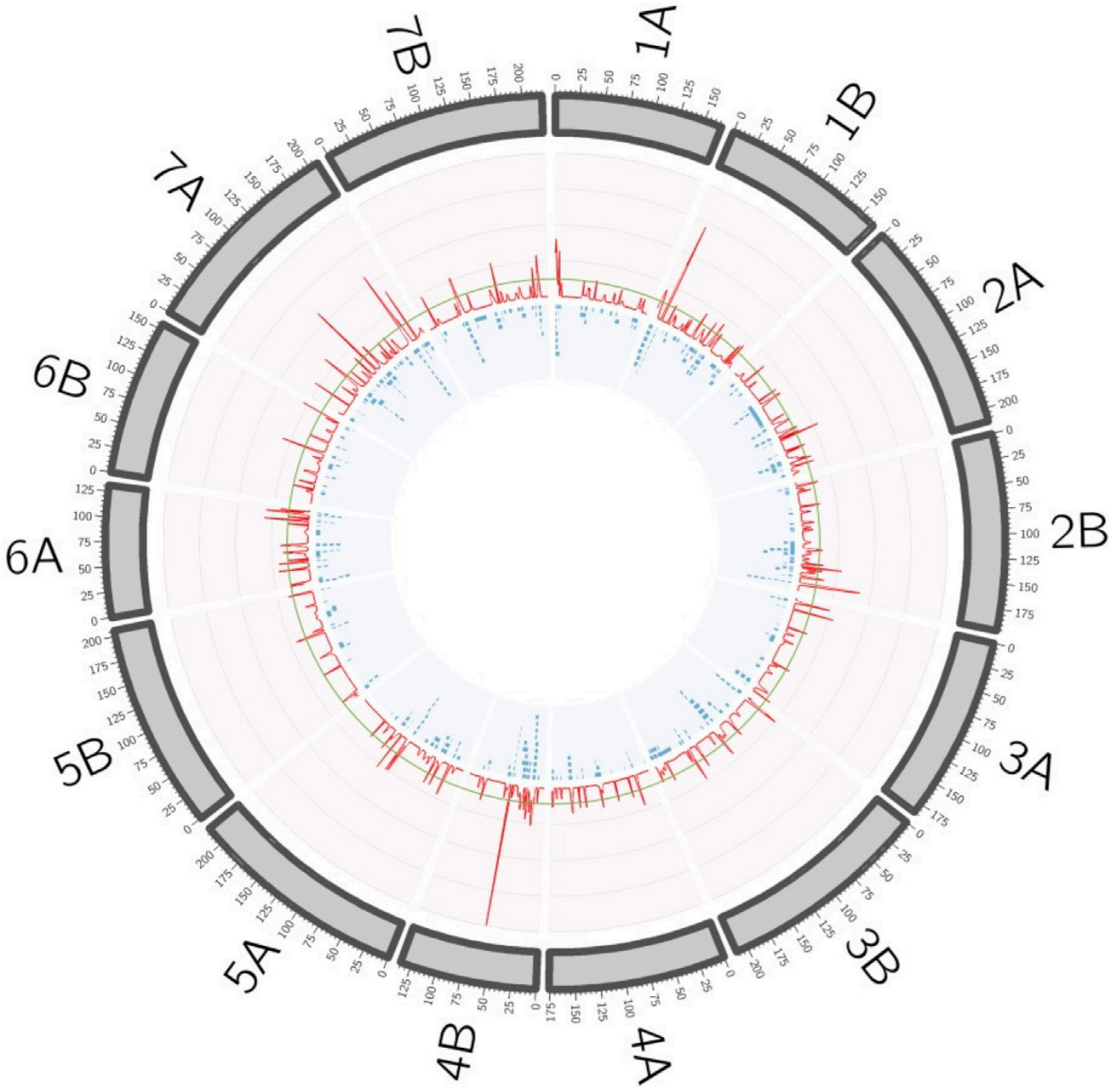
QTL hotspots defined by the overview density index (red line). Blue boxes represent the projected MTAs on the durum wheat consensus map.

In order to simplify the MTA information, QTL hotspots were defined using the QTL overview density index defined by Chardon et al. (2004) for each centimeter of the durum wheat consensus map. A total of 564 peaks were identified using the mean of the overview index across the 14 chromosomes (0.15) as the threshold, whereas using a high threshold (0.75), a total of 158 peaks were detected (Figure 2). These 158 peaks were reduced to 92 QTL hotspots (Supplementary Table S2), 47 in genome A and 45 in genome B. QTL hotspots were selected based on three criteria for candidate gene identification. First, hotspots with a physical distance lower than 20 Mb were identified, and later only those containing three or more MTA with a PVE higher or equal to 0.04 were chosen. As a result, 20 QTL hotspots including 121 MTA with a PVE mean of 0.11 were selected (Table 3). These hotspots were considered as breeding QTL.

**TABLE 3:** Selected QTL hotspots. PVE refers to the mean of all the MTA in the hotspot.

QTL	Chr	CI (cM)	CI Svevo (Mb)	CI CS (Mb)	N MTA	PVE	Traits
1	1A	1–7	1.1–7.6	1.3–7.6	11	0.07	Color, DEE, DOE, FIRM, GPS, GS, PPO, SV, WTS, YPC
4	1B	2–5	6.3–8.2	6.0–9.6	7	0.08	CWT, FIRM, DOE, GPC, GS, MIXO, SV
5	1B	15–16	19.5–19.6	22.2–22.9	8	0.07	CWT, DEE, DOE, DOTE, GPS, GS, MIXO, SV
10	1B	117–118	628.2–632.4	633.3–637.8	3	0.13	b*, GPC, YPC
15	2A	119	586.6–596.9	593.5–603.6	3	0.19	L*, YPC
26	2B	182–184	775.7–777.5	787.8–789.6	7	0.10	DEE, dif, GPC, GLUT, PRLOSS, VIT, WG
31	3B	5–6	5.8–15.2	6.2–13.9	5	0.06	CLOSS, CWT, FIRM, WTS, YPC
33	3B	87–88	503.1–512.0	491.4–507.0	7	0.06	Color, CWT, dif, FIRM, GS, MIXO, YPC
35	3B	145–146	749.3–752.6	739.2–742.1	3	0.12	HMW-GS/LMW-GS, YPC
47	4B	19–21	19.1–22.1	19.7–22.6	6	0.14	CLOSS, DOE, GPS, PLOSS, VIT, YPC
49	4B	29–30	26.6–27.0	28.1–28.5	3	0.36	PLOSS, YPC
51	4B	39–41	59.1–75.6	60.5–77.0	5	0.12	b*, TW, YPC
52	4B	59–60	473.1–492.7	469.0–488.7	8	0.11	GPC, PLOSS, PRLOSS, WG, YPC
57	5A	111–115	489.4–503.6	526.4–540.6	7	0.19	CWT, FIRM, GLI, Gli + Glu, WTS
64	6A	1–4	4.7–7.4	6.6–9.5	5	0.04	Color, GL, GLUT, IP, YPC
73	6A	125–129	603.5–615.3	609.1–616.3	6	0.05	AX CLOSS, Color, dif
79	7A	59–61	60.9–63.6	63.1–65.5	7	0.09	GPC, SPROT, VIT, WG, YPC
81	7A	102	148.3–159.4	151.4–161.7	5	0.06	DEE, DOTE, GPC, GPS, YPC
85	7A	179–184	691.0–699.0	697.0–705.1	10	0.12	AX, Color, CWT, dif, FIRM, WTS, YPC
92	7B	204–206	714.7–716.2	734.3–741.2	5	0.09	IP, YPC

The comparison of the breeding QTL coverage in the genetic and physical maps resulted in different distribution along chromosomes. Consensus genetic and physical chromosomes were defined based on the differentiation of five bins per chromosome according to its genetic and physical distance respectively, and each bin corresponding to 1/5 of the total length. Whereas for the genetic chromosome the QTL hotspots were equally distributed in each one of the bins (with 16% on bin 3, 21% on bins 1, 2, and 5, and 22% on bin 4), for the physical chromosome and unequal distribution was observed, with the higher number of QTL hotspots on telomeric bins (83% on bins 1 and 5, 12% on bin 4, and 2 and 3% on bins 2 and 3, respectively) (Figure 1D).

### Synteny among species and MQTL candidate genes identification

**FIGURE 3:**
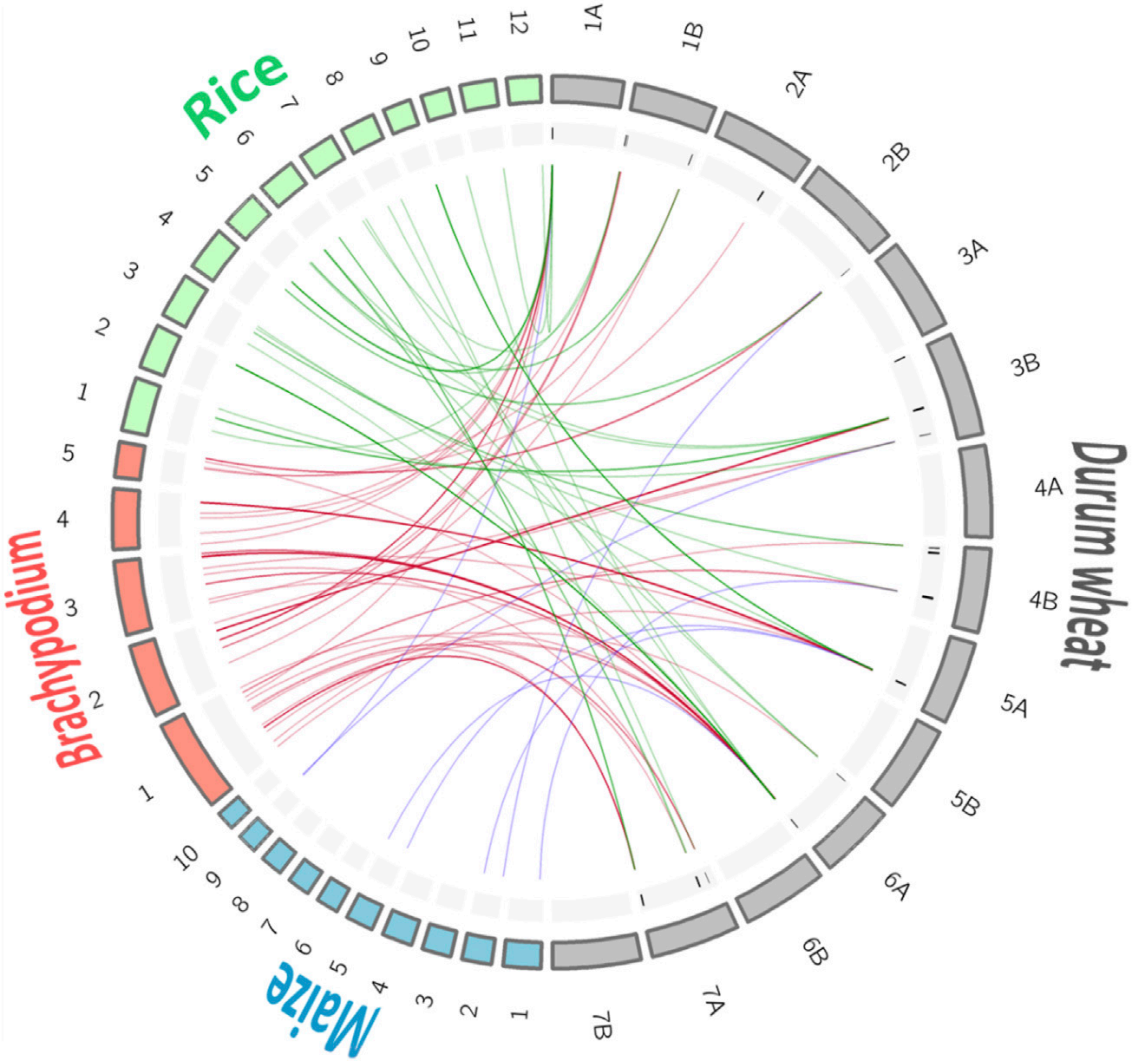
Synteny among genomes for the selected quality QTL hotspots. For better visualization of the chromosome links the size of the *Brachypodium* and rice chromosomes is multiplied by 10.

Thanks to the pipeline described in Materials and Method, it was possible to identify syntenic genome regions between the *Triticum* spp., markers associated with the breeding QTL on three genomes (*Brachypodium*, *Z. mays,* and *O. sativa*) (Figure 3; Table 4; Supplementary Table S3).

**TABLE 4:** Synteny among species in the QTL hotspot regions. bd, *Brachypodium distachyum*; os, *Oryza sativa*; zm, *Zea mays*.

Chromosome	Hotspot	Chromosome name							
1A	qhotspot1	bd1	bd2	bd4	os1	os5	os11	os12	zm9
1B	qhotspot4	bd2	os5	os7					
1B	qhotspot5	bd4	bd2						
1B	qhotspot10	bd2	bd5	os5					
2A	qhotspot15	bd5							
2B	qhotspot26	bd5	os4	zm2					
3B	qhotspot33	bd2	os1	os5	os6				
3B	qhotspot35	bd2	bd3	os1	zm9				
4B	qhotspot47	bd1	os3						
4B	qhotspot52	bd1	os3	zm1					
5A	qhotspot57	bd1	bd3	bd4	os3	os9	zm2	zm5	
6A	qhotspot64	bd1	os6	os7					
6A	qhotspot73	bd1	bd3	bd5	os2	os3	os8	os10	zm5
7A	qhotspot79	bd1	bd3	os8					
7A	qhotspot81	bd1	os6						
7A	qhotspot85	bd1	os6						
7B	qhotspot92	bd3							

A total of 134 markers from the breeding QTL were identified in syntenic regions (ortho-MQTL) of the *Brachypodium* (78), rice (49), and maize (7) genomes. Chromosome 6A showed the highest number of markers with syntenic regions with other genomes, whereas chromosomes 3A, 4A, 5B, 6B, and 7B did not show any homologous marker. Genome A represented 70% of the syntenic markers.

Using the genomic sequences located in the breeding QTL from Svevo, *Z. mays, O. sativa*, and *B. distachyum*, candidate genes correlated to ortho-MQTL were identified (Table 5).

**TABLE 5:** Genes identified through meta-QTL analysis in each qhotspot and detected in the following databases: bread wheat cultivar Chinese Spring (CS) genome sequence, durum wheat cultivar Svevo genome sequence, Brachypodium db, Rice db, and MaizeGDB.

Traits	QTL hotspot	Chrom	Chinese spring	Svevo	*Brachypodium*	*Oryza sativa*	*Zea mays*
Color, DEE, DOE, FIRM, GPC, GS, PPO, SV, WTS, YPC	1	1A	Gamma gliadin-A1, A3, A4, and LMW-A2 gene	HMW glutenin A gene	LMW glutenin subunit (GLU-3)	ATPase, AAA family domain	Paired amphipathic helix protein Sin3-like 3
CWT, FIRM, DOE, GPC, GS, MIXO, SV	4	1B	—	Transforming growth factor-beta receptor-associated protein 1 G	Methionine S-methyltransferase	Methionine S-methyltransferase	Methionine S-methyltransferase
CWT, DEE, DOE, DOTE, GPS, GS, MIXO, SV	5	1B	Gamma gliadin-B1, B2, B4, B6, delta gliadin-B1, omega gliadin-B3, B6, LMW-B2, and LMW-B3 genes	—	Pectin acetylesterase 5	—	—
b*, GPC, YPC	10	1B	Cysteine protease	Dynamin-related protein	F-box/FBD/LRR-repeat protein	Dynamin family protein	—
L*, YPC	15	2A	Transcription factor Y subunit B-3-like	—	Leucine-rich repeat-containing G-protein coupled receptor 4	—	—
DEE, dif, GPC, GLUT, PRLOSS, VIT, WG	26	2B	Potassium ion transport	—	K(+) efflux antiporter 2	Potassium efflux antiporter	K(+) efflux antiporter 2 chloroplastic
Color, CWT, dif, FIRM, GS, MIXO, YPC	33	3B	Endonuclease activity	Evolutionarily conserved C-terminal region 2	Glucan 1,3-beta-glucosidase A	Selenium-binding protein	—
HMW-GS/LMW-GS, YPC	35	3B	Golgi transport complex	—	Potassium transporter 2	Potassium transporter	Component of oligomeric Golgi complex 4
CLOSS, DOE, GPS, PLOSS, VIT, YPC	47	4B	Acid-amino acid ligase activity	—	Inositol hexakisphosphate and diphosphoinositol-pentakisphosphate kinase VIP2	WD repeat-containing protein	—
GPC, PLOSS, PRLOSS, WG, YPC	52	4B	Transmembrane 9 superfamily member 3-like	Ethylene response factor 1 (ERF1)	Transmembrane 9 superfamily member 3	Transmembrane 9 superfamily member	Transmembrane 9 superfamily member
CWT, FIRM, GLI, Gli + Glu, WTS	57	5A	Phosphorelay response regulator activity	Protein PAT1 1 G	Protein PAT1 homolog	Glycosyl transferase family 8	Adenylate kinase
Color, GL, GLUT, IP, YPC	64	6A	Putative nitric oxide synthase	—	Ubiquitin carboxyl-terminal hydrolase 2	Ubiquitin protein	—
AX CLOSS, Color, dif	73	6A	ABC transporter F family member 3	—	ABC transporter F family	ABC transporter	ABC transporter F family member 3
GPC, SPROT, VIT, WG, YPC	79	7A	Cysteine synthase-like	—	Cysteine synthase	Cysteine synthase	-
DEE, DOTE, GPC, GPS, YPC	81	7A	Cell surface glycoprotein 1-like	—	Pyrophosphate--fructose 6-phosphate 1-phosphotransferase subunit beta 1	WD domain, G-beta repeat domain	—
AX, Color, CWT, dif, FIRM, WTS, YPC	85	7A	Serine/Threonine-Protein Kinase	—	Serine/threonine protein phosphatase	Ser/Thr protein phosphatase	—
IP, YPC	92	7B	Sucrose synthase 7-like	Sucrose synthase	Sucrose synthase 7	—	—

The investigation of collinear regions within the five genomes resulted in the identification of 17 synthenic regions containing paralogous genes with similar functions that can be considered as promising candidate genes controlling the quality trait considered (Table 5). On qhotspot1, which was correlated to the dough extensibility, firmness, grain protein content, and gluten index and strength, the genes gamma gliadin-A1, A3, A4, and LMW-A2, HMW glutenin A gene, and LMW glutenin subunit (GLU-3) were identified on Chinese Spring, Svevo and *Brachypodium*, respectively confirming the correlation between the genomic region considered and the traits control. Instead, two different genes were identified in the same qhotspot1 for rice and maize (ATPase, AAA family domain, and paired amphipathic helix protein Sin3-like 3, respectively).

On qhotspot4.qhotspot10, qhotspot15, qhotspot35, qhotspot47, qhotspot52, qhotspot64, and qhotspot81 were identified in Svevo, maize, rice, and *Brachypodium* genes for biological processes, such as DNA replication, cell wall development, and secondary metabolite production, with no direct correlation with the traits corresponding to the QTL regions (Table 5). No colinear genes were detected on qhotspot5 between the species analyzed, but candidate genes correlated to the dough extensibility and tenacity, grain protein content, gluten index, and strength (gamma gliadin-B1, B2, B4, B6, delta gliadin-B1, omega gliadin-B3, B6, LMW-B2, and LMW-B3 genes) were detected in Svevo.

Qhotspot26, qhotspot73, qhotspot79, qhotspot85, and qhotspot92 showed the same genes for all the genomes considered, involved in biological process, but not directly correlated with the traits considered, except for qhotspot92, which showed a correlation between trait IP and *sucrose synthase* genes identified in Chinese Spring, Svevo and *Brachypodium*. The other two correlations were detected between traits and genes on qhotspot33 and qhotspot57 in *Brachypudium* and rice, respectively, but no collinearity was found between species.

## Discussion

The detection of major loci for quantitative traits (QTL) is a key tool of modern genetics to identify candidate genes controlling polygenic traits. Kernel quality traits are often quantitative, so controlled by multiple genes/QTL, strongly influencing the end-product quality of commercial wheat varieties, and determining the type of products that can be produced. Thus, it is possible to distinguish a commercial value at the beginning of wheat production and a technological value linked to the worldwide market requirements for end-product uses (Colasuonno et al., 2021). The development of new genotypes with high-quality value is an important tool for breeders besides industrial and consumer requirements. (Colasuonno et al., 2021).

The study of quantitative traits is complex and required the identification of molecular markers and/or genes tightly linked to quality traits to be used in Marker Assisted Selection (MAS) programs. A modern approach based on GWAS, and meta-QTL analysis allowed the evaluation of wide collections identifying numerous new genes/QTL and the comparison of results through meta-analysis. In the present study, we provide a wide study for the evaluation of genetic loci controlling kernel quality traits in durum wheat through the identification of new stable genome-wide QTL hotspots, and correlation through a synteny analysis between different species including *Z. mays*, *B. distachyon*, and *O. sativa*.

The study has been focused on 10 GWAS studies reporting 57 quality traits recorded in wide collections and representative of durum wheat genetic variability for protein content and composition (HMW, LMW), yellow color, fiber, grain microelement content such as iron, magnesium, potassium, selenium, sulfur, calcium, cadmium, kernel vitreousness, semolina, and dough quality test (all reported in Table 1). A total of 395 MTA, equally distributed on the A and B genomes, were used in genome-wide QTL hotspot detection. In order to include all the MTA in a single map they were projected on the durum wheat consensus map developed by Maccaferri et al. (2015) as described by Chardon et al. (2004) and Colasuonno et al. (2021), estimating the CIs, not reported in the original studies, according to the linkage disequilibrium (LD) decay for each chromosome (as reported in materials and method). 97% of the MTAs reported a CI lower than 10 cm, with a phenotypic variance explained (PVE) lower than 0.1, these values are in accordance with those reported by Soriano and Alvaro (2019), Soriano et al. (2021) for quantitative inheritance.

In order to reduce the number of MTA and simplify the information, 92 QTL hotspots (47 in genome A and 45 in genome B) were identified using the QTL overview density index defined by Chardon et al. (2004). Some features were observed when comparing the distribution pattern of QTL hotspots in the genetic and physical maps. Chromosomes were divided into five bins (genetic and physical) and percentage of QTL hotspots per bin was calculated (as it is reported in the Material and Methods section). While QTL hotspots were equally distributed along the genetic chromosomes, a different distribution was observed for the physical chromosomes with higher number of QTL hotspots on telomeric bins (83% on bins 1 and 5). This is in accordance with what was reported by several authors in QTL mapping, which observed that QTL are highly abundant in some genomic regions, and, frequently, correlated traits are clustered closely together in some specific loci as compared to others (Goffinet and Gerber, 2000; Schadt et al., 2003; Chardon et al., 2004; West et al., 2007; Breitling et al., 2008; Wu et al., 2008; Wang et al., 2014; Basnet et al., 2015; Martinez et al., 2016; Yang et al., 2019).

A clustering between QTL and traits has also been reported in classical MQTL analysis by Soriano et al. (2021) that grouped 318 QTL including quality, biotic and abiotic traits in 85 MQTL with number of traits involved in each MQTL ranging from 1 to 12, and the number of QTL per MQTL ranged from 2 to 11. This phenomenon may have several causes, including QTL with high allelic polymorphisms and interesting pleiotropic effect or closely linked QTL controlling correlated traits and frequently co-localized in the same regions (Zhao et al., 2011; Vuong et al., 2015; Mengistu et al., 2016; Zhang et al., 2020). As the QTL hotspots can lead to the identification of genes that affect the traits of interest, and further help to build networks among QTL hotspots, genes, and traits, the qhotspot detection analysis at genome-wide level has been a key step toward deciphering the genetic architectures of quantitative traits in genes, genomes, and genetics studies (Breitling et al., 2008; Fu et al., 2009; Neto et al., 2012; Wang et al., 2014; Yang et al., 2019; Wu et al., 2021).

Previous studies identified ortho-MQTL for yield-related traits and nitrogen use efficiency (Saini et al., 2021; Singh et al., 2022). According to these authors, the conserved nature of the ortho-MQTL suggests that may be associated with regulatory elements affecting many genes.

In this study, we used the breeding QTL for the investigation of collinear regions between durum and bread wheat, with *Brachypodium*, maize, and rice.

The candidate gene investigation allowed the correlation between some ortho-MQTL with genes, such as for qhotspot1 which showed collinearity between Chinese Spring, Svevo, and *Brachypodium*, a clear association with DOE, FIRM, GPC, GS, and genes for gliadin and glutenin. A similar association was investigated on qhotspot5 where it was detected on the Chinese Spring genome again genes for gliadin and glutenin associated with DOE, DOTE, GPS, and GS. Both these associations are in accordance with data reported in the literature which located genes encoding glutenin and gliadin on chromosome groups 1 (*Gli-A1*, *Gli-B1*, *Gli-D1*, *Glu-A1*, *Glu-B1*, *Glu-D1*, *Glu-A3*, *Glu-B3*, and *Glu-D3* loci) and 6 (*Gli-A2*, *Gli*-*B2,* and *Gli-D2* loci) (Zaefizadeh et al., 2010; Dubois et al., 2016; Utebayev et al., 2019; Asri et al., 2021).

Another association was detected on qhotspot26, which was mainly related to protein content traits and showed co-location with potassium ion transport in wheat. In fact, the influence of potassium transport efficiency in kernel protein content has been largely studied and reported in the literature (Beaton and Sekhon, 1985). Analyzing the collinearity with the other genomes used in this study, the syntheny with rice, maize, and *Brachypodium* was also detected.

An important correlation was determined on qhotspot92 between the gliadins (IP traits) and the gene *S. synthase*, which was found in Chinese Spring, Svevo, and *Bachypodium* genome sequences. The association between this trait and the gene was already highlighted by Marín-Sanz et al. (2022) who reported that genes implicated in starch synthesis, including *S. synthase*, were up-or down-regulated in RNAi lines characterized by a strong reduction in α/β-, ω-, and γ-gliadins. All the other association qhotspot/genes corresponded to genes involved in biological processes, mainly regulatory or transport genes.

## Conclusion

This study aimed to a better understanding of the genetic architecture controlling quality traits in durum wheat. GWAS is considered a powerful and complementary tool to QTL analysis for the detection of genes directly correlated to phenotypic traits. However, a meta-analysis of GWAS permits to identify consensus regions among different germplasm controlling different traits and regulatory genes which for some of them play a major role in the control of the trait per se. Comparative genomics analysis among bread and durum wheat, rice, maize, and *Brachypodium* allowed the identification of the conserved orthologous set of DNA sequences for candidate genes underpinning quantitative traits. The strict relation between comparative genomics and gene identification emphasizes the importance of the orthologous genes identified, opening the possibility to transfer information across species.

## Data Availability

The original contributions presented in the study are included in the article/Supplementary Material. Further inquiries can be directed to the corresponding authors.
